# Micronutrient deficiencies among children and women in Bangladesh: progress and challenges

**DOI:** 10.1017/jns.2016.39

**Published:** 2017-01-03

**Authors:** Faruk Ahmed, Noreen Prendiville, Anuradha Narayan

**Affiliations:** 1 Public Health, School of Medicine and Menzies Health Institute Queensland, Griffith University, Gold Coast Campus, QLD 4220, Australia; 2 UNICEF, Kampala, Uganda; 3 UNICEF, Dhaka, Bangladesh

**Keywords:** Micronutrients, Anaemia, Intervention programmes, Policy, Bangladesh, BDHS, Bangladesh Demographic and Health Survey, IDA, Fe-deficiency anaemia, IFA, Fe–folic acid, NMS 2011–2012, National Micronutrients Status Survey 2011–2012, MNP, multiple micronutrient powder, NPNL, non-pregnant and non-lactating, VAD, vitamin A deficiency

## Abstract

This paper provides a comprehensive review of the current situation regarding micronutrient deficiencies among children and women in Bangladesh. This review also discusses the successes and current challenges of existing intervention programmes. Data from nationally representative and selected small surveys since the 1980s that have reported on the status of at least one micronutrient in children and/or women have been examined. National policy documents/reports on existing interventions have been analysed. While the severity of various micronutrient deficiencies has declined since the 1980s, a significant proportion of preschool-age children remains with deficiencies in vitamin A (20·5 %), Zn (44·5 %) and vitamin D (39·6 %); about one-third of these children are anaemic, and 10·7 % of the children are Fe deficient. A high proportion of non-pregnant and non-lactating women is deficient in Zn (57 %) and I (42 %), while one-quarter of women live with anaemia and vitamin B_12_ and vitamin D (21 %) deficiencies. Nearly one-half of the pregnant and lactating women are anaemic. Suboptimal diets, poor hygiene, infection and infestation are identified as some of the key factors associated with high levels of deficiencies. Multiple approaches and interventions are being supported, and while some notable progress has been achieved, significant challenges continue, including those related to coverage, quality and compliance. It is concluded that although current intervention programmes have made some progress in controlling the severe deficiencies, micronutrient deficiencies in Bangladesh remain a considerable problem. More well-integrated approaches for strengthening the existing intervention programmes are needed. In addition, new intervention strategies for alleviating and preventing specific micronutrient deficiencies are recommended.

Micronutrient deficiency is widely prevalent throughout the world, affecting more than two billion people^(^^1^^)^. However, the scale of the problem is much greater in low- and middle-income countries where multiple micronutrient deficiencies often present concomitantly as a result of diets with limited diversity, poor bioavailability and limited micronutrient content, in addition to poor hygiene and infections^(^^2^^)^. Micronutrient deficiencies (i.e. vitamin A and Zn deficiencies, in particular) have been estimated to account for one million child deaths per year, and Fe deficiency alone is responsible for 115 000 maternal deaths per year^(^^3^^)^. In addition to an increased risk of mortality, micronutrient deficiencies are associated with impaired resistance to infections, decreased nutrient uptake and delayed or impaired physical, mental and psychomotor development^(^^4^^,^^5^^)^. Therefore, these deficiencies affect people's quality of life, which can have an impact on productivity^(^^1^^,^^6^^)^. The importance of reducing micronutrient deficiencies in children and women is well recognised. For example, a systematic review of vitamin A supplementation trials in children aged 6–59 months showed a 24 % reduction in all-cause mortality and a 28 % reduction in diarrhoea-related mortality^(^^7^^)^. Another review showed that preventive Zn supplementation in children younger than 5 years reduced diarrhoea by 13 % and pneumonia by 19 %^(^^8^^)^. More recently, a systemic review of multiple micronutrient supplementation during pregnancy showed a significant reduction in low birth weight, small-for-gestation and preterm births^(^^9^^)^.

In Bangladesh, the prevalence of undernutrition has been decreased significantly and is likely to achieve the nutrition Millennium Development Goal^(^^10^^)^. Although several strategies have been implemented over the past decades to address the situation, the prevalence of micronutrient deficiency still remains very high and is considered a significant public health problem^(^^11^^)^. The recently concluded National Micronutrients Status Survey 2011–2012 (NMS 2011–2012)^(^^11^^)^, together with the analysis and discussions related to the development of a strategy to address micronutrient deficiencies, has presented an opportunity to examine the current micronutrient status among children and women in the country and review the successes and challenges of existing interventions. This paper also discusses the proposed intervention strategies for the prevention and control of micronutrient deficiencies in Bangladesh.

## Methodology

This review examined all of the available nationally representative surveys since the 1980s that have reported on the prevalence of at least one micronutrient deficiency in children (infants, preschool-age children, school-age children and/or adolescents) and/or women (non-pregnant and non-lactating (NPNL), pregnant and/or lactating women). Data from some small studies were used if national data were not available. Reports on the effectiveness of existing intervention programmes and various national policy documents have been analysed. National surveys and policy documents were searched online using official websites of relevant research institutes/organisations. In addition, the lead research institutes/organisations were consulted to obtain documents that were not available online. The national surveys that did not report on the prevalence of anaemia and/or micronutrient deficiency in children and/or women were excluded. Because of the limited data on Fe, Zn and vitamin B_12_ deficiencies, selected non-representative studies were also included in the analyses.

## Current situation of micronutrient deficiency

This section focuses on the extent and nature of micronutrient deficiencies among children and women in the country and is followed by an analysis of the factors contributing to the deficiency. The literature search identified nine national surveys and four nutrition surveillance reports that met the inclusion criteria. Of the included studies, four reported on the prevalence of vitamin A deficiency (VAD), five on the prevalence of anaemia and three on the prevalence of I deficiency only. Only one study reported on the prevalence of anaemia and a range of micronutrient deficiencies. The details of the methods and prevalence of various micronutrient deficiencies of the referenced survey reports/articles are summarised in Table 1.

**Table 1.** Description of studies and the prevalence of anaemia and micronutrient deficiencies in children and women in Bangladesh

Micronutrients	Reference	Study period	Study design	Sampling method	Sample size	Prevalence of deficiency
Anaemia	(19)	1997–1998	National survey, rural population	Multi-stage cluster sampling	Children 6–59 months (*n* 1199) NPNL women (*n* 1082) Pregnant women (*n* 120) Lactating women (*n* 694)	Children 6–59 months, 47 % NPNL women, 45 % Pregnant women, 49·2 % Lactating women, 48·7 %
	(20)	2003	National survey, urban population	Two-stage stratified cluster sampling	Children 6–59 months (*n* 861) Adolescent females (*n* 723) NPNL women (*n* 884) Pregnant women (*n* 500) Lactating women (*n* 665)	Children 6–59 months, 55·7 % Adolescent females, 28·7 % NPNL women, 32·9 % Pregnant women, 40·5 % Lactating women, 36·1 %
	(23)	2001	National surveillance project, rural population	Multi-stage cluster sampling	Pregnant women (*n* 108)	46·7 %
	(24)	2004	National surveillance project, rural population	Multi-stage cluster sampling	Pregnant women (*n* 102)	38·8 %
	(21)	2011	National survey	Two-stage cluster sampling	Preschool children (*n* 2353) NPNL women (*n* 3973) Pregnant women (*n* 347) Lactating women (*n* 1356)	51 % 40 % 49·6 % 47·8 %
	(11)	2011–2012	National survey	Three-stage cluster sampling	Preschool children (*n* 600) School children (*n* 1500) NPNL women (*n* 1050)	Preschool children, 33·1 % Children 6–11 years, 19·1 % Children 12–14 years, 17·1 % NPNL women, 26 %
Fe deficiency	(25)	1996	Survey, peri-urban schools	Convenience sampling	Adolescent girls 11–16 years (*n* 548)	17 %
	(26)	1998	Baseline survey, garment factory	Convenience sampling	Adolescent girls 14–19 years (*n* 289)	67–86 %
	(11)	2011–2012	National survey	Three-stage cluster sampling	Preschool children (*n* 600) School children (*n* 1500) NPNL women (*n* 1050)	Preschool children, 10·7 % Children 6–11 years, 3·9 % Children 12–14 years, 9·5 % NPNL women, 7·1 %
I deficiency	(18)	1993	National survey	Three-stage cluster sampling	Goitre survey: School children (*n* 12 862) NPNL women (*n* 17 210). Urinary I: School children (*n* 528) NPNL women(*n* 4518)	Goitre: School children, 50 % NPNL women, 55·6 % I deficiency: School children, 70·7 % NPNL women, 67·4 %
	(16)	1999	National survey	Cluster sampling	School children 5–11 years (*n* 8929) NPNL women 15–44 years (*n* 12 049)	Goitre: School children, 17·2 % NPNL women, 18·2 % I deficiency: School children, 42·5 % NPNL women, 45·6 %
	(17)	2004–2005	National survey	Cluster sampling	Goitre survey: School children (*n* 6400) NPNL women (*n* 2447). Urinary I: School children (*n* 2400) NPNL women (*n* 2401)	Goitre: School children, 6·2 % NPNL women, 11·7 % I deficiency: School children, 33·8 % NPNL women, 38·6 %
	(11)	2011–2012	National survey	Three-stage cluster sampling	Urinary I: School children (*n* 1350) NPNL women (*n* 1500)	I deficiency: School of children, 40 % NPNL women, 42·1 %
Zn deficiency	(37)	2002	Population-based study, rural area	Convenience sampling	Pregnant women (*n* 740)	55 %
	(38)	2003	Community-based study, rural area	Convenience sampling	Infants (*n* 1066)	57 %
	(11)	2011–2012	National survey	Three-stage cluster sampling	Preschool children (*n* 1050); NPNL women (*n* 1500)	Preschool children, 44·6 % NPNL women, 57·3 %
Vitamin A deficiency	(14)	1997–1998	National survey	Multi-stage cluster sampling	Night blindness survey: Children 6–59 months (*n* 26 868) NPNL women (*n* 6493) Pregnant women (*n* 2434) Lactating women (*n* 13 984) VAD: Children 6–59 months (*n* 1136) NPNL women (*n* 380) Pregnant women (*n* 118) Lactating women (*n* 687)	Night blindness: Children 6–59 months, 0·62 % NPNL women, 1·7 % Pregnant women, 2·7 % Lactating women, 2·4 % VAD: Children 6–59 months, 21·7 % NPNL women, 5 % Pregnant women, 23·7 % Lactating women, 14 %
	(12)	1982–1983	National survey	Multi-stage cluster sampling	Preschool children Rural (*n* 18 660) Urban (*n* 3675)	Night blindness: Preschool children Urban: 2·8 % Rural : 3·8 %
	(11)	2011–2012	National survey	Three-stage cluster sampling	Preschool children (*n* 1200) School children (*n* 1500) NPNL women (*n* 1050)	VAD: Children 6–59 months, 20·9 % NPNL women, 5·4 %
Vitamin B_12_ deficiency	(37)	2002	Population-based study, rural area	Convenience sampling	Pregnant women (*n* 740)	46 %
	(38)	2003	Community-based study	Convenience sampling	Infants (*n* 1066)	31 %
	(11)	2011–2012	National survey	Three-stage cluster sampling	NPNL women (*n* 1050)	23 %
Folate deficiency	(11)	2011–2012	National survey	Three-stage cluster sampling	NPNL women (*n* 1050)	9·1 %
Vitamin D deficiency	(11)	2011–2012	National survey	Three-stage cluster sampling	Preschool children (*n* 461) School children (*n* 557) NPNL women (*n* 613)	Serum vitamin D <50·0 nmol/l: Preschool children, 39·6 % School children, 45·5 % NPNL women, 71·5 % Serum vitamin D <25·0 nmol/l: Preschool children, 7·5 % School children, 6·5 % NPNL women, 21·0 %

NPNL, non-pregnant and non-lactating; VAD, vitamin A deficiency (serum retinol <0·7 µmol/l).

### Vitamin A deficiency

VAD has been identified as a serious public health problem in Bangladesh since the 1960s. Although the severity of the problem has decreased remarkably, the prevalence of VAD (defined by the WHO as a serum retinol concentration <0·70 µmol/l) has remained the same over the past decade, especially among preschool-age (6–59 months) and school-age children^(^^11^^)^. Currently, 20·5 % of the preschool-age children are suffering from VAD, with the highest prevalence among children living in the slums (e.g. 38 % in preschool-age children and 27 % in school-age children)^(^^11^^)^. While there are no recent estimates available on the severe forms of VAD, previous surveys indicated that the prevalence of night blindness in preschool-age children has declined remarkably over the past two decades^(^^12^^,^^13^^)^; it was 3·7 % in 1982–1983^(^^12^^)^ and dropped to 0·04 % in 2005^(^^13^^)^.

The recent NMS 2011–2012 reported only a 5·4 % prevalence of VAD among NPNL women^(^^11^^)^, which is similar to the prevalence reported in a 1997–1998 National Vitamin A Survey^(^^14^^)^. However, nearly one-third of the NPNL women have a suboptimal vitamin A status (i.e. serum retinol concentration <1·05 µmol/l)^(^^11^^)^. No current estimates are available on the prevalence of VAD among pregnant and lactating women.

Low socio-economic status, being a slum dweller, low levels of knowledge of vitamin A-rich food and/or their health benefits, poor intake of animal sources of vitamin A especially by households with severe food insecurity and predominant share of plant-based vitamin A with low bioavailability have been identified as factors associated with VAD among all population groups^(^^11^^,^^15^^)^.

### Iodine deficiency

The current prevalence of I deficiency, based on urinary I concentration <100 µg/l, in school-age children is as high as 40 %^(^^11^^)^, reflecting a figure similar to the 42·5 % reported in 1999^(^^16^^)^ and an increase from the 33·8 % reported in 2004–2005 (Table 1)^(^^17^^)^. There are no estimates on the current prevalence of goitres (a severe form of I deficiency) in school-age children. However, previous surveys indicated a substantial reduction in goitre prevalence in school-age children from 50 % in 1993^(^^18^^)^ to 17·2 % in 1999^(^^16^^)^ to 6·2 % in 2004–2005^(^^17^^)^.

The NMS 2011–2012 reported a 42 % prevalence of I deficiency, based on urinary I level, in NPNL women^(^^11^^)^, which was 45·6 % in 1999^(^^16^^)^ and 38·6 % in 2004–2005^(^^17^^)^. The National Iodine Deficiency Disorder Survey in 2004–2005 reported goitre rates of 11·7 and 14·6 % in NPNL women and pregnant women, respectively^(^^17^^)^, with the higher risk of I deficiency among women either living in rural areas and/or belonging to lower socio-economic groups^(^^11^^)^.

Household food insecurity, lack of access to iodised packaged salt, rural residency, low levels of awareness about the health benefits of I and iodised salts, consumption of industrial salt (non-iodised) and lack of preservation knowledge about iodised salts have been identified as the major risk factors of I deficiency in the Bangladeshi population^(^^15^^)^.

### Anaemia and iron deficiency

According to the NMS 2011–2012, the prevalence of anaemia, defined by an Hb concentration <120 g/l in NPNL women and <110 g/l in children aged 6–59 months, affects 33 % of children aged 6–59 months and 26 % of NPNL women^(^^11^^)^. These percentages reflect a significant decrease in the prevalence reported in 1997–1998 (47 % in children aged 6–59 months and 45 % in NPNL women)^(^^19^^)^ and in 2003 (55·7 % in children aged 6–59 months and 32·9 % in NPNL women)^(^^20^^)^. However, the Bangladesh Demographic and Health Survey (BDHS) conducted in 2011 found higher levels, with a 51 % prevalence of anaemia in preschool-age children and a 40 % prevalence in NPNL women^(^^21^^)^. The NMS 2011–2012^(^^11^^)^ and BDHS 2011^(^^21^^)^ were conducted within a 1-year period, but the differences in prevalence are probably attributed to methodological differences. To measure Hb levels, the BDHS used capillary blood samples by finger prick, whereas the NMS 2011–2012 used venous blood samples. Capillary blood samples often produce inconsistent results (blood drawing requires extreme care and training), whereas venous blood samples produce more reliable results. However, it is clear that a significant proportion of preschool-age children and NPNL women are still anaemic. While there are no recent data, earlier surveys have indicated a 30–40 % prevalence of anaemia among adolescent girls^(^^19^^–^^20^^,^^22^^)^. Further, according to the BDHS in 2011, approximately one-half of the pregnant (49·6 %) and lactating (48 %) women are also anaemic^(^^21^^)^. In 2001, the prevalence of anaemia in pregnant women was 47 %^(^^23^^)^, but it was 39 % in 2004^(^^24^^)^, reflecting an increase in the current prevalence among pregnant women.

Earlier small-scale studies indicated that Fe deficiency is a major cause of anaemia^(^^25^^,^^26^^)^, but the results from the NMS 2011–2012 suggest that the national prevalence of Fe deficiency (serum ferritin concentration <120 µg/l in preschool-age children and <150 µg/l in NPNL women) is only 10·7 % in preschool-age children and 7·1 % in NPNL women^(^^11^^)^. Further, only 7·2 % of the preschool-age children and 4·8 % of the NPNL women reported suffering from Fe-deficiency anaemia (IDA). IDA was defined by combined cut-offs for an Hb concentration <110 g/l and serum ferritin <120 µg/l in preschool-age children and an Hb concentration <120 g/l and serum ferritin <150 µg/l in NPNL women^(^^11^^)^. Unfortunately, there are no data on Fe deficiency and IDA among pregnant women. The prevalence of IDA is even lower in school-age children^(^^11^^)^, indicating that Fe deficiency may not be the major cause of anaemia in the Bangladeshi population. While the NMS 2011–2012 reports that dietary Fe provides only 41–82 % of the RDA across different population groups^(^^11^^)^, Fe content in groundwater appears to be high in some parts of Bangladesh^(^^27^^)^, predominantly in the ferrous (Fe^2+^) form, which is more bioavailable than the ferric form^(^^27^^,^^28^^)^. The low prevalence of Fe deficiency in the NMS 2011–2012 was attributed to the high Fe concentration in drinking water from tube-wells^(^^11^^)^. Of note, a small-scale study in northern rural Bangladesh reports a positive association of daily Fe intake from drinking water with plasma ferritin and total body Fe in women^(^^29^^)^. However, a recent article based on NMS 2011–2012 data reported a differential prevalence of anaemia and Fe deficiency in Bangladeshi NPNL women living in areas of high and low Fe in groundwater^(^^30^^)^.

Yet, as anaemia levels remain high in children and women, less than 10 % can be explained by IDA. Other contributors, such as deficiencies in vitamin B_6_, vitamin B_12_, vitamin A, vitamin C, folic acid and riboflavin^(^^31^^)^, need to be examined, reinforcing the role that other haematopoietic micronutrients play in controlling and preventing 90–95 % of anaemia cases. Reviewing non-nutritional contributors to anaemia (i.e. malaria, worm infestation, chronic infections and genetic disorders (e.g. haemoglobinopathies))^(^^32^^–^^34^^)^, it is noted that the risk of malaria is generally low in Bangladesh. While there are no national data on the prevalence of thalassaemia, it is considered a likely contributor, with one study reporting a 28 % prevalence of thalassaemia and an associated risk of anaemia^(^^35^^)^.

### Zinc deficiency

The NMS 2011–2012 reports a 44·6 % prevalence of Zn deficiency among preschool-age children and 57·3 % prevalence in NPNL women, with the highest rate among those living in the slums^(^^11^^)^. Zn deficiency was defined by a serum concentration <9·9 mmol/l in preschool-age children and a serum concentration <10·1 mmol/l in NPNL women according to the International Zinc Consultative Group^(^^36^^)^. It is important to note that serum Zn is homeostatically regulated and unable to detect marginal deficiency; therefore, a high prevalence of low serum Zn is considered to be a reasonable indicator of a relatively severe deficiency^(^^36^^)^. Although there are no national level data on infants and pregnant women, two small-scale studies in rural communities report a 55 % prevalence of Zn deficiency in pregnant women^(^^37^^)^ and a 57 % prevalence of Zn deficiency in infants^(^^38^^)^. Low socio-economic status, household food insecurity, low intake of animal sources of Zn and high intake of plant-based diet with a very high content of phytate (an inhibitor of Zn absorption) are the main drivers behind poor Zn nutrition^(15)^.

### Vitamin D deficiency

Based on a serum vitamin D level <25 nmol/l, 7·5 % of preschool-age children and 6·5 % of school-age children are deficient in vitamin D^(^^11^^)^. The NMS 2011–2012 indicates that the prevalence of vitamin D deficiency in preschool-age children is the highest among the poorest and the severely food-insecure households; however, for school-age children, the prevalence of vitamin D deficiency is the highest among the richest and the food-secure households^(^^11^^)^. In addition, 32·1 % of preschool-age children and 39·0 % of school-age children are living with an insufficient vitamin D status (i.e. serum vitamin D <50·0 nmol/l), with the highest prevalence among children living in the slums^(^^11^^)^. In 2008, the National Rickets Survey revealed a 1 % prevalence of rickets among children between the ages of 1 and 15 years^(^^39^^)^.

The figures for NPNL women suggest that 21 % of these women are deficient based on a serum vitamin D level <25·0 nmol/l, and 50 % of them have an insufficient vitamin D status (serum vitamin D level <50 nmol/l)^(^^11^^)^. No corresponding national-level data are available for pregnant women.

### B vitamin deficiencies

To date, no data are available on the national level estimates of B vitamin deficiencies among infants and children. However, in rural Bangladesh, one earlier small study has shown a 31 % prevalence of vitamin B_12_ deficiency in infants^(^^38^^)^; whereas another study has shown a 25 % prevalence in folate deficiency, a 7 % prevalence of vitamin B_12_ deficiency and an 89 % prevalence of vitamin B_2_ deficiency among adolescent school girls who are anaemic^(^^40^^)^.

According to the NMS 2011–2012, 9 % of NPNL women are deficient in folate, and 23 % of these women have some degree of vitamin B_12_ deficiency^(^^11^^)^. One small-scale study has shown a 46 % prevalence of vitamin B_12_ deficiency in women in early pregnancy^(^^37^^)^.

## Aetiology of micronutrient deficiency

A large majority of Bangladeshi people follow a diet consisting of predominantly plant-based foods. They have a lack of dietary diversity with a minimum amount of animal food, including eggs, milk and milk products. Thus, a poor-quality diet with poor bioavailability is potentially the major contributor to micronutrient deficiencies in the country^(^^41^^,^^42^^)^. Although there appears to be an overall increase in the consumption of animal foods in the country (26·2 g/capita per d in 2010^(^^43^^)^ *v*. 20·8 g/capita per d in 2005^(^^44^^)^), the majority of the population has an inadequate intake for a range of micronutrients. A study on the dietary micronutrient intake among young children and their primary female caregivers in rural Bangladesh indicates a very low overall mean prevalence of adequacy of micronutrient intake, based on estimated average requirements, in children (43 %) and women (26 %)^(^^45^^)^. For children, the prevalence of adequate intake was <30 % for Fe, riboflavin and vitamin B_12_, and it was <10 % for Ca, folate and vitamin A^(^^45^^)^. A very similar picture was also presented in the NMS 2011–2012^(^^11^^)^. Furthermore, based on the Household Income and Expenditure Survey 2010, 35 % of the population reportedly has mean dietary diversity scores lower than six out of twelve food groups, and thus, is considered to be at risk of micronutrient deficiency^(^^43^^)^.

In addition to a poor-quality diet, the major underlying causes of micronutrient deficiency in the country have been reported to be limited diversity due to low socio-economic status and household food insecurity, low levels of understanding in relation to an optimal diet and hygiene practices, along with infection and infestation^(^^15^^)^. It is noteworthy that infectious disease and micronutrient deficiencies exacerbate one another in a vicious cycle. Infections deplete micronutrients, and with limited stores to draw upon, the immune system weakens further and becomes less capable of fighting the infection^(^^46^^)^.

## Current policies and intervention programmes

The nature and progress of the current policies and programmes that have been developed to alleviate the problem of micronutrient deficiencies in the country were reviewed to develop the subsequent analysis and discussion related to understanding the persistent deficiencies and moving towards recommendations on new directions. Table 2 shows the current intervention programmes and their coverage rates for prevention and control of micronutrient deficiencies in Bangladesh.

**Table 2.** Current intervention programmes for the prevention and control of various micronutrient deficiencies in Bangladesh

Intervention programme	Target group	Current coverage rate	Comments
Vitamin A supplementation	Children under 5 years	Infants aged 6–11 months: 85·4 % Children aged 12–59 months: 93·7 %	No remarkable variation in coverage between urban and rural areas
Fortification of edible oil with vitamin A	Whole population	Not available	Recently initiated, no monitoring data available
Fe–folate supplementation	Pregnant women/adolescent girls and non-pregnant women	Pregnant women: received IFA through ANC: around 60 %	ANC care is weak – less than 30 % of women attend ANC in the first trimester, and attendance at 4+ visits is around 30 %. Inadequate supply at ANC or inadequate knowledge and practice of the providers – very weak counselling. Poor monitoring. No nationwide programme for adolescent girls and non-pregnant women
Universal salt iodisation	Whole population	Adequately iodised salt (>20 ppm): Retailer's level: 66·4 % Household level: 57·6 %	Coverage is lowest in rural areas due to the availability of non-iodised salts which are less costly
Home fortification with MNP	Children under 5 years	Adherence to MNP*: 70 %	A small study conducted in an intensive programme area
Promotion of diversified diet	Whole population	N/A	Policies are in place to improve diversified agriculture, fisheries and aquaculture and livestock development, and community-based nutrition programmes, but much stronger efforts are needed to achieve goal

IFA, Fe–folic acid; ANC, antenatal care; ppm, parts per million; MNP, multiple micronutrient powder; N/A, not applicable.

* Adherence was defined by the consumption of one MNP sachet per d in the past 60 d.

### Programmes aimed at improving infant and young child feeding practices

Through a very wide range of means and methods, the government of Bangladesh, UN agencies, non-governmental organisations and donors have supported interventions aimed at improving infant and young child feeding (IYCF) practices from birth, when exclusive breastfeeding is promoted, through the various stages of the introduction of complementary food from 6 months and beyond. In a number of cases, these interventions are supported together with interventions related to health, food, livelihoods, water, sanitation and hygiene.

### Anaemia-control programme for children aged 6–59 months

The deworming of children aged 24–59 months with albendazole is part of the country's anaemia-control programme for children and is included during the twice-yearly, vitamin A supplementation campaign. In 2011, the national coverage rate for deworming was 77 %^(^^47^^)^.

To prevent anaemia and other micronutrient deficiencies, the National Strategy for the Prevention and Control of Anaemia in Bangladesh has recommended using multiple micronutrient powder (MNP) in the diet of children between 6 and 23 months old (and between 24 and 59 months old if resources are available)^(^^48^^)^. Currently, there is one large-scale area-based MNP programme in place supported by the Bangladesh Rural Advancement Committee (BRAC)/Global Alliance for Improved Nutrition (GAIN) that uses the government-approved five-component (i.e. Fe, folic acid, Zn and vitamins A and C) powder. In 2013, the community health workers (Shasthya Shebika) of BRAC distributed 14·5 million sachets of MNP in rural communities through home visits^(^^49^^)^.

### Vitamin A supplementation

In settings where VAD is a public health problem, 6-monthly vitamin A supplementation is strongly recommended by the WHO for infants and children under 5 years of age as a public health intervention to reduce child morbidity and mortality^(^^50^^)^. Bangladesh initiated this programme in 1973. Coverage increased from 56 % in 1993^(^^51^^)^ to 80 % in 1999^(^^52^^)^ and is reported to be approximately 90 % since 2006^(^^53^^)^.

While the WHO does not recommend vitamin A supplementation in postpartum women as a public health intervention^(^^54^^)^ due to a lack of evidence of its benefits, the Bangladesh government recommends postpartum vitamin A supplementation. The coverage of postpartum vitamin A supplementation remains low at approximately 36 %^(^^55^^)^.

### Fortification of edible oil with vitamin A

Bangladesh initiated the fortification of edible oil with vitamin A in December 2011. Out of twenty-two edible-oil refineries, sixteen produce vitamin A-fortified edible oil. While the government endorsed the ‘National Edible Oil Fortification Law 2013’, ensuring the 100 % fortification of refined edible oil with vitamin A, this programme has not yet reached its targets in relation to coverage^(^^15^^)^.

### Universal salt iodisation programme

In Bangladesh, the Universal Salt Iodisation (USI) programme has been in place for more than two decades. By 1999, while nearly 99 % of the edible salts at the factory level were iodised, not all edible salts were adequately iodised according to defined standards^(^^56^^)^. The current coverage of adequately iodised salt (≥20 parts per million (ppm)) at the retailer level is 66·4 % and at the household level is 57·6 %^(^^11^^)^. The coverage is lowest in rural areas. Currently, about a one-quarter of households buy non-iodised bulk salt, and 84 % of those who do not consume iodised salt cite the relatively higher price as the main reason^(^^11^^)^.

The USI programme continues to face challenges, including delays in revising the law to include the iodisation of salt destined for the food industry and animals, as per guidance from the WHO^(^^57^^)^. Other significant challenges include insufficient enforcement at the production and retailer level; inadequate information on the actual production of edible and industrial salts; lack of access to packaged, fortified salt in rural and hard-to-reach areas; higher prices of iodised salt; absence of quality control on the importation of edible salt; and overall weak monitoring systems. Despite significant investments in mass communication and targeted information campaigns, consumer and retailer interest remains relatively low^(^^15^^)^.

### Iron–folic acid supplementation

While the NMS 2011–2012 indicates overall low Fe deficiency and IDA in children and NPNL women in Bangladesh, an Fe–folic acid (IFA) supplementation programme for pregnant women has been implemented for several decades aimed at preventing and managing Fe deficiency and anaemia. This programme provides IFA supplements (60 mg Fe and 400 µg folic acid daily) to pregnant women from the second trimester until 90 d after delivery. The IFA supplementation is one of the components of the Health, Population and Nutrition Sector Development Program (HPNSDP). The programme was implemented by the Directorate General of Family Planning (DGFP) and delivered by the Family Welfare Visitors of the DGFP as part of the antenatal care services at the Satellite Clinics and Maternal and Child Welfare Centres.

However, apart from a number of areas that receive direct support from development partners and non-governmental organisations, coverage remains suboptimal, with a low proportion of women consuming the full recommended dosage. The 2011 report on the State of Food Security and Nutrition in Bangladesh indicated that 37 % of women did not consume any IFA supplements during pregnancy^(^^47^^)^, and among those who took the IFA supplements, more than one-half started in the third trimester^(^^47^^)^. Coverage rates vary widely by geographical area and by place of residence, with compliance higher among urban-dwelling women. While antenatal care coverage has increased dramatically from 31 % in 1993^(^^51^^)^ to 79 % in 2014^(^^58^^)^, only 31 % of pregnant women attended the recommended minimum four visits in 2014. The Bangladesh Health, Population and Nutrition Sector Development Program (HPNSDP) sets a target of 50 % of the pregnant women making at least four visits by 2016^(^^59^^)^, and clearly Bangladesh is far behind from meeting this target. However, one recent report that conducted an analysis of how well antenatal care clinics are distributing the IFA tablets showed that only 60 % of the pregnant women attended at least one antenatal care, but 26 % of them did not receive any IFA tablets. This could be due to an inadequate supply or inadequate knowledge and practice of the providers^(^^60^^)^.

### Iron–folic acid supplementation for non-pregnant and non-lactating women and adolescent girls

In Bangladesh, twice-weekly supplementation with 60 mg Fe and 400 µg folic acid is recommended for the prevention and treatment of IDA in adolescent girls and NPNL women^(^^52^^)^. From 2005 to 2010, a school-based programme in areas outside Dhaka aimed to distribute weekly IFA supplements to adolescent girls. In addition, adolescent girls, newlywed women and postpartum women were provided IFA supplements and anthelmintic treatment in areas covered by the National Nutrition Programme. In 2011, the National Nutrition Programme was replaced by the National Nutrition Services as one of the operational plans under the Health, Population and Nutrition Sector Development Program (HPNSDP) and implemented by the Institute of Public Health Nutrition to ensure the mainstreaming of nutrition. While the National Nutrition Services has established a guideline for IFA supplementation for NPNL women and adolescent girls, currently there is no nationwide IFA supplementation programme or platform to reach this target group.

The current IFA supplementation programme has several strengths that include a government-approved national anaemia strategy, IFA distribution guidelines and an integrated health and family planning wing under the National Nutrition Services operational plan. However, one major weakness is the lack of a detailed plan of action (roadmap), along with inadequate coordination and monitoring between the Ministry of Health and Family Welfare and other non-governmental organisations that are also delivering IFA tablets through their programmes^(^^60^^)^. An analysis of the bottlenecks leading to the inadequacy of effective coverage in relation to IFA supplementation undertaken by UNICEF in 2012–2013 showed that very specific issues needed to be addressed relating to supply, uptake of services and compliance with treatment at the household level. Specific targets for IFA supplementation are often absent and not adequately covered by certain indicators, such as attendance for antenatal care (UNICEF, Dhaka: unpublished results). Counselling and follow-up related to compliance with supplementation regimens are not routinely included in care protocols.

The level of education of the beneficiaries and lack of awareness about the benefit of IFA supplements are some of the key contributing factors to the low coverage rate^(^^15^^)^. Ensuring a consistent and adequate supply of IFA tablets and introduction of indicators and targets related to actual bottlenecks will be required to address the current inadequate coverage.

### Promotion of diversified diet

Throughout Bangladesh, many government and non-government entities have supported a wide variety of approaches and programmes aimed at increasing the diversity and quality of the diet. The Bangladesh Country Investment Plan is described as a ‘roadmap towards investment in agriculture, food security and nutrition’, and identified twelve programmes as part of its Plan of Action 2008–2015^(^^61^^)^. The Bangladesh Country Investment Plan supports community-based homestead gardening, rearing small livestock, aquaculture and awareness building, aiming to increase household availability and access to food in general, but especially micronutrient-rich foods. The 2011–2012 food security nutritional surveillance programme report estimated that 42 % of households had both homestead gardens and poultry, 14 % had homestead gardens alone, 20 % had poultry and 24 % had neither^(^^47^^)^.

To optimise the potential of these programmes to address micronutrient deficiencies, targeting, outcome monitoring and stronger communication interventions will be required. Although the National Nutrition Services supports mass media campaigns, social mobilisation and behaviour change communication activities at the health facility and community clinic levels, these specific nutrition-related messages also need to be reinforced through the Ministry of Agriculture and other food-related ministries. Opportunities exist to create and strengthen markets for nutritious foods, as well as to invest in value chain and postharvest technologies that can help retain and enhance the nutritional content of foods.

## Programme implications

Using the findings of the NMS 2011–2012 and complementary data, the government and partners developed the ‘National Strategy on Prevention and Control of Micronutrient Deficiencies, Bangladesh (2015–2024)’, which was approved in May 2015^(^^15^^)^. This strategy has laid out a very comprehensive action plan divided into six strategic areas: (i) policy, guidelines and legislation; (ii) intervention programmes; (iii) partnerships and coordination; (iv) capacity building; (v) advocacy and communication; and (vi) monitoring, evaluation and research^(^^15^^)^.

In this strategy document, a number of key interventions are recommended for controlling micronutrient deficiencies. Some of these interventions have been implemented for many years and mainly require strengthening, while other interventions are new in the country and require adaptation to country context and system development. These interventions are divided into three broad categories. The first is ensuring that all individuals, throughout their life-cycle, have a fully adequate nutrient intake, and maintain optimal health, through optimal infant and young child feeding (IYCF) and improved dietary diversification. Second, to complement diet- and care-based approaches, evidence-based and cost-effective food fortification is recommended, with the provision that the fortified foods must reach those who most need them. Finally, to address the immediate requirements of individuals and populations most at risk or already experiencing the debilitating and life-threatening effects of micronutrient deficiencies, targeted supplementation is recommended.

## Discussion

The most recent national survey, NMS 2011–2012, has provided critical information to inform decision making and programming related to public health and nutrition in Bangladesh. It has also presented a number of challenges at both the technical and operational levels. While the country moved ahead to develop a new strategy, a number of issues are worthy of further discussion, analysis and research.

First, it is noteworthy that despite very high coverage of a 6-monthly vitamin A capsule supplementation programme for children aged 6–59 months in the country, currently one in five children are living with VAD, with the highest prevalence among children living in the slums. Several studies have demonstrated that a 6-monthly high-dose vitamin A capsule can only achieve a transient and small increase in serum retinol that could sustain the level for about 2 months. Therefore, this programme is unable to protect against mild–moderate VAD (serum retinol <0·70 µmol/l) up to 6 months, although it can protect the eyes from clinical VAD^(^^62^^,^^63^^)^. A recent article by Mason *et al*.^(^^64^^)^ indicated that frequent intakes of vitamin A in physiological doses either through regular low-dose supplementation or through food-based approaches, including fortification, can be highly effective in increasing serum retinol, thus reducing VAD. The authors emphasised the need of a policy shift from periodic vitamin A capsules to increasing frequent regular intakes of vitamin A at physiological levels^(^^64^^)^. However, the replacement of vitamin A capsules with a food-based strategy is not an immediately feasible option in the absence of access to adequate vitamin A-rich food, including fortified food for those who are vulnerable.

Second, recent findings on the association between the natural Fe content of groundwater and Fe status have introduced new challenges for public health programming. It is evident that there is variability in the prevalence of Fe deficiency across the country due to varied Fe levels in groundwater^(^^30^^,^^65^^)^. This may have serious implications for a future IFA supplementation programme for controlling anaemia and Fe deficiency in pregnant women in the country and thus there is an urgent need for reviewing the current IFA supplementation guidelines. However, it is important to recognise the fact that the need for Fe increases significantly during pregnancy to meet the demand of physiological changes and fetal development^(^^66^^)^. Based on the data in NPNL women, it is difficult to predict whether or not Bangladeshi pregnant women living in areas of either high or low Fe in groundwater can maintain a satisfactory Fe status, which is absolutely critical for fetal development, throughout pregnancy without IFA supplementation. On the other hand, it is also equally important to know whether or not IFA supplementation in this population, especially those living in areas of high Fe in groundwater, can cause Fe overload. Fe is a strong pro-oxidant and high body Fe levels have been linked to oxidative stress that in turn has been implicated in atherosclerosis, CVD, diabetes and the metabolic syndrome^(^^67^^,^^68^^)^. Elevated Fe stores are also associated with the exaggeration of systemic infections^(^^69^^)^. Further, excess dietary intake of Fe has been found to be associated with increased growth and virulence of enteric pathogens which in turn increase gut inflammation^(^^70^^,^^71^^)^. IFA supplementation during pregnancy is a key direct nutrition intervention that UNICEF is supporting the government to scale-up to effective coverage; however, given the recent findings of the NMS 2011–2012, the relevancy of this intervention needs further investigation. The persistence of non-Fe-related anaemia also requires further understanding.

Third, given the variability in the prevalence of Fe deficiency and IDA in the country based on the most recent micronutrient survey, MNP programmes for children aged 6–59 months should be carefully evaluated to see if there is a need for change in the composition of MNP.

Fourth, the USI programme in Bangladesh provides a good example of an area where we know exactly what needs to be done; however, despite the investment of significant resources and an apparent good commitment for implementation, achievement has been lower than expected. This might also be a moment to comment on the fact that while many programmes are consistently increasing their coverage (in numbers), it is not adequately intensive to keep pace with the rapid population growth; therefore, percentages remain stagnant.

Fifth, it is important to note that the current prevalence of Zn deficiency is much higher than VAD in children and NPNL women in the country. Currently, the Bangladesh government has implemented an adjunctive treatment of Zn with oral rehydration in diarrhoea for young children, which is independent of the underlying Zn status of the population. Thus, there is a need to consider the appropriate intervention for the prevention of Zn deficiency in the country.

Finally, in Bangladesh and globally, we have a debate related to short/medium-term approaches *v*. long-term approaches to address micronutrient deficiencies. It will be important in the months and years ahead to monitor the situation closely, ensuring that the appropriate mix of interventions is supported using evidence-based approaches. Approaches that address immediate threats to wellbeing should not be seen to compete with meaningful interventions to ensure the long-term dietary adequacy of the population. It will be increasingly important to identify specific nutrition-related indicators in food-related programmes, ensuring that interventions first understand and subsequently address specific dietary deficits, be they seasonal or for specific groups of people (e.g. age or wealth group). In the short term and to meet the specific needs of vulnerable groups, agriculture and horticulture programmes that enhance diet quality and diversity must be combined with supplementation programmes specifically designed to address dietary deficits.

## Conclusion

This review illustrates that while the severity of many micronutrient deficiencies in Bangladesh has decreased remarkably over the past few decades, a significant proportion of the population, especially children and women, is still deficient in critical micronutrients. Several intervention programmes are in place to alleviate the problem of micronutrient deficiencies, especially for vitamin A, Fe and I deficiency. However, the success of these interventions is far from satisfactory. Given the complex nature of factors contributing to micronutrient deficiencies in Bangladesh, like other low-income countries, the prevention and control of micronutrient deficiency in the country are unlikely to be achieved through nutrient-specific nutrition policies and programmes. More holistic approaches that can integrate into short- to long-term strategies are necessary to achieve and maintain sustainable improvement of the present situation. It is also essential that there are sufficient resources and collaboration between health and food sector workers, which is vital for ensuring quality service delivery and accurate monitoring and reporting for improved outcomes in relation to the micronutrient status of targeted groups. Finally, advocacy is needed to influence government policy across core sectors in combating micronutrient malnutrition through support from the highest level of leadership.
